# Rapid identification of capsular serotype K1/K2 *Klebsiella pneumoniae* in pus samples from liver abscess patients and positive blood culture samples from bacteremia cases via an immunochromatographic strip assay

**DOI:** 10.1186/s13099-019-0285-x

**Published:** 2019-02-22

**Authors:** Ching-Hsun Wang, Po-Liang Lu, Esther Yip-Mei Liu, Yih-Yuan Chen, Fu-Mei Lin, Yi-Tsung Lin, Feng-Yee Chang, Jung-Chung Lin

**Affiliations:** 1 Division of Infectious Diseases and Tropical Medicine, Department of Internal Medicine, Tri-Service General Hospital, National Defense Medical Center, Taipei, Taiwan, ROC; 2 Department of Internal Medicine, Kaohsiung Medical University Hospital, Kaohsiung City, Taiwan, ROC; 3 College of Medicine, Kaohsiung Medical University, Kaohsiung City, Taiwan, ROC; 4 Department of Biochemical Science and Technology, National Chiayi University, Chiai-Yi, Taiwan, ROC; 5 Institute of Infectious Diseases and Vaccinology, National Health Research Institutes, Miaoli, Taiwan, ROC; 6 Division of Infectious Diseases, Department of Medicine, Taipei Veterans General Hospital, Taipei, Taiwan, ROC; 7 School of Medicine, National Yang-Ming University, Taipei, Taiwan, ROC

**Keywords:** *Klebsiella pneumoniae*, Serotypes K1, Serotype K2, Serum agglutination, PCR, ICS

## Abstract

**Background:**

In Asia, serotype K1/K2 *Klebsiella pneumoniae* are the major capsular serotypes that cause liver abscess or bacteremia in patients. The purpose of this study was to compare novel immunochromatographic strips (ICSs), which can rapidly detect *K. pneumoniae* serotypes K1/K2 in clinical samples, to conventional capsular serotyping methods.

**Methods:**

Pus drainage samples from 16 patients with a liver abscess caused by *K. pneumoniae*, blood samples from 112 positive flagged blood culture bottle and a subsequent single colony in the medium were tested with the ICS. The results were then compared to findings of capsular swelling tests. Samples subjected to the polymerase chain reaction (PCR) analysis were used as reference.

**Results:**

The identification of *K. pneumoniae* via the traditional bacterial culture from pus samples took 3.4 days on average (ranging from 2.2 to 5.5 days). Further capsular serotyping of *K. pneumoniae* by the capsular swelling test of pure isolates lasted 5–10 min, and the PCR method took ~ 4 h. As for ICSs, the time for direct identification of the *K. pneumoniae* capsular serotype K1/K2 in pus was < 4 min (ranging from 2 to 4 min). The results of ICSs were consistent with capsular swelling tests and PCR methods. Testing of 112 blood culture samples and subsequent single colonies in the medium with ICSs yielded consistent results for most samples.

**Conclusions:**

This study indicates that ICSs can rapidly detect *K. pneumoniae* serotypes K1 and K2 in pus or positive flagged blood culture broth samples within 5 min. Their accuracy is comparable to that of the conventional capsular serotyping methods such as a serum agglutination assay or PCR.

## Background

Pyogenic liver abscesses caused by *Klebsiella pneumoniae* constitute an emerging global infectious disease as reported recently [1]. Among 79 serotypes of the capsular polysaccharides of *K. pneumoniae*, serotypes K1/K2 have the highest prevalence in many southeast Asian countries and are associated with virulent types in *K. pneumoniae*-induced bacteremia and liver abscesses, particularly in cases with metastatic endophthalmitis [2–4]. Appropriate early antibiotic treatment—with the aim of decreasing mortality and morbidity related to pyogenic liver abscesses caused by *K. pneumoniae*—is an important clinical task. It usually takes at least 3 days to isolate *K. pneumoniae* from pus and identify it via blood cultures in the traditional clinical laboratory setting, and serotyping is not routinely performed. The capsular swelling test via anti-capsular type sera, countercurrent immunoelectrophoresis from pure isolates, or polymerase chain reactions (PCRs) have been used to identify *K. pneumoniae* capsular serotypes. They take hours to days when bacterial cultures or direct clinical samples are used [5, 6]. Although the direct detection of *K. pneumoniae* capsular serotypes in clinical samples is possibly based on the PCR assay, false negatives may sometimes occur due to interference from a non purified DNA samples. Preparation of purified DNA may be needed to increase the accuracy of detection in a PCR assay.

In a previous study, we have evaluated a colloidal-gold-based immunochromatographic strip (ICS) kit for the rapid detection of *K. pneumoniae* serotypes K1 and K2 [7]. In the current study, we aimed to test the efficacy of this kit for detecting *K. pneumoniae* capsular serotypes directly in pus drainage samples, positive blood culture samples, and pure bacterial colonies.

## Results

### Comparison of different testing results on the capsular serotyping of K1 and K2 of *K. pneumoniae* isolates

A total of 16 patients with a liver abscess caused by *K. pneumoniae*, as confirmed by radiographic and microbiological analyses, were enrolled in this study. The demographic characteristics (age, gender, comorbid diabetes, duration of symptoms/signs, lobes and size of a liver abscess, duration of identification by PCR or strip test, and serotypes) of the patients are shown in Table 1.

**Table 1:** Demographic data and capsular serotype identification of enrolled patients

Case	Sex/age	DM	Duration of S/S (days)	Lobe of abscess	Size of abscess (cm)	Identification time:		Serotypes confirmed by:		
						Culture (days)	ICS (minutes)	Colony capsular swelling test	Colony PCR	Pus ICS
1	F/56	Yes	5	Left	5.5	3.1	4	K55	Non-K1/K2	Non-K1/K2
2	F/62	Yes	6	Right	6	5	3	K7	Non-K1/K2	Non-K1/K2
3	F/65	No	3	Left	5.1	2.5	3	K1	K1	K1
4	F/45	No	1	Left	4.4	3.5	3	K2	K2	K2
5	F/62	No	5	Right	8.6	2.6	3	K1	K1	K1^a^
6	M/41	Yes	9	Right	9.6	4.2	3	K1	K1	K1
7	M/60	No	5	Right	5.5	4	3	K2	K2	K2
8	M/51	No	6	Right	6.3	2.2	3	K2	K2	K2
9	M/88	No	5	Right	9.8	3.7	4	K53	Non-K1/K2	Non-K1/K2
10	M/42	Yes	5	Right	5	4.2	2	K14	Non-K1/K2	Non-K1/K2
11	F/84	Yes	3	Right	4.2	3.9	4	K1	K1	K1^a^
12	F/74	Yes	2	Right	2	5.5	4	K57	Non-K1/K2	Non-K1/K2
13	M/61	No	2	Right	7.7	2.8	2	K1	K1	K1
14	M/74	Yes	7	Right	7.4	2.4	3	K1	K1	K1
15	M/61	Yes	14	Right	7.1	2.8	3	K1	K1	K1
16	M/29	No	5	Right	6	2.5	3	K1	K1	K1

*M* male, *F* female, *DM* diabetes mellitus, *S/S* symptoms and signs, *ICS* immunochromatographic strip, *PCR* polymerase chain reaction

^a^Endophthalmitis was found after being confirmed to have serotype K1 by the strip test

There were nine males (56.3%, 9/16) and seven females (43.7%, 7/16) among the 16 patients with *K. pneumoniae* liver abscesses. Comorbid diabetes mellitus was present in 50.0% of the patients (8/16). The average diagnostic time from the start of the symptoms/signs of the liver abscess in clinical settings was 5.2 days (ranging from 1 to 14 days). The most common lobe with a liver abscess was on the right side (~ 81.3% or 13/16). The average size of a liver abscess was 6.26 cm in diameter (ranging from 2 to 9.8 cm). The duration of *K. pneumoniae* identification using the traditional bacteria culture for pus was 3.4 days (ranging from 2.2 to 5.5 days). Starting from a culture colony, the duration of identification of *K. pneumoniae* capsular serotypes by the capsular swelling test and PCR was demonstrated to require an additional 5–10 min and 4 h, respectively. For the rapid strip test, the time for identification of *K. pneumoniae* capsular serotype K1/K2 directly in pus was only 3.1 min (ranging from 2 to 4 min).

**Fig. 1:**
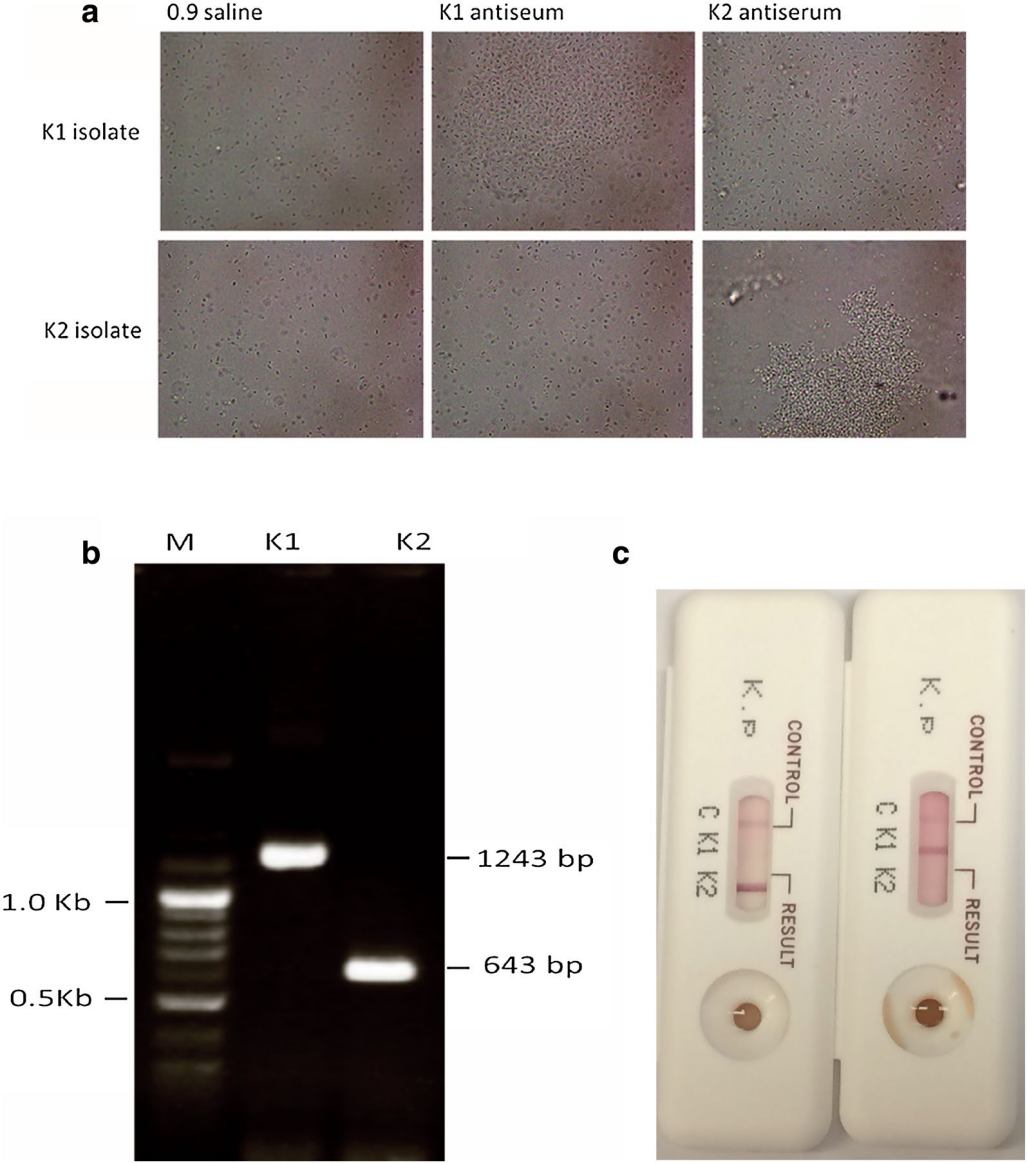
The results of serotypes K1 and K2 *K. pneumoniae* identification by different typing methods. **a** Capsular swelling test showing capsular serotypes K1/K2 following reaction with K1 and K2 antiserum. Saline (0.9%) was used as control. Images were captured using a phase contrast microscope (original magnification, 1000×). **b** Capsular serotypes K1/K2 detected by the PCR testing. Visualization of the bands on an agarose (1.5%) gel for the capsular serotypes K1/K2 strains (K1, K2). Size of each amplicon is indicated at the side. M, markers. **c** Capsular serotypes K1/K2 detected by means of ICSs. The red line in the ICS indicated serotype K1 or K2 is shown

**Fig. 2:**
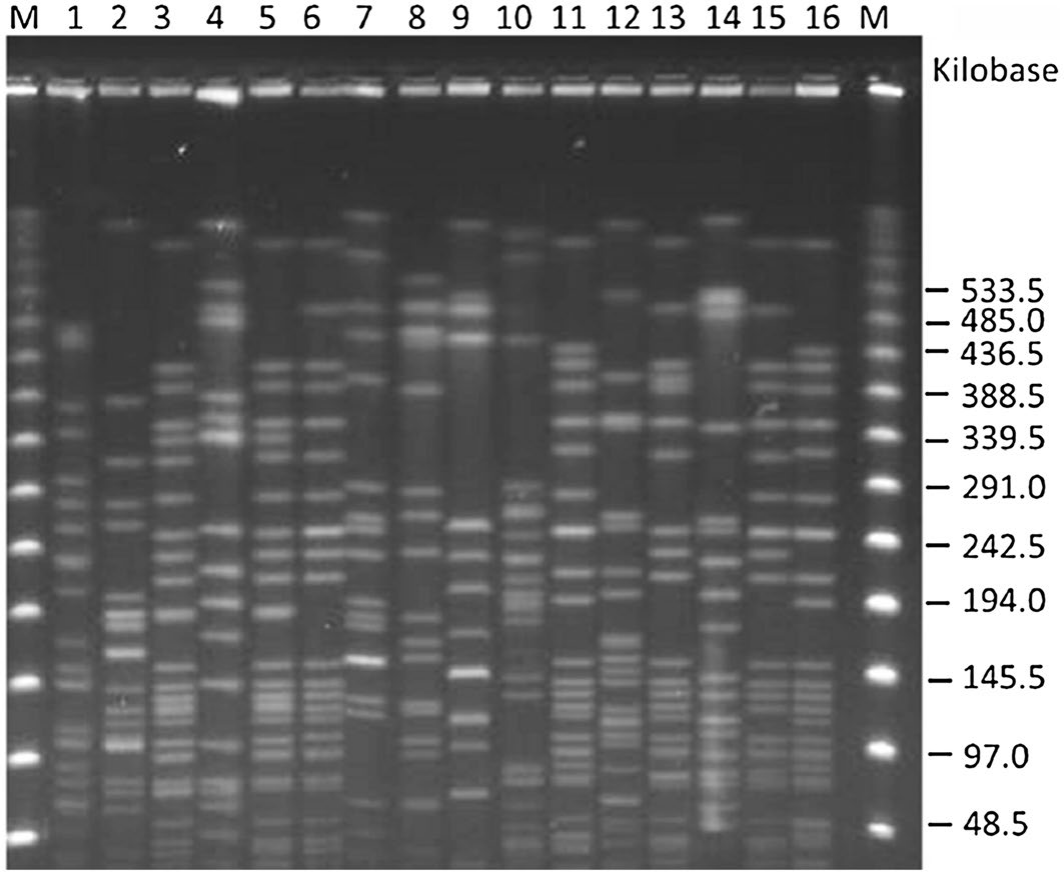
Pulsed-field gel electrophoresis (PFGE) analysis of *K. pneumoniae* strains isolated from 16 enrolled patients. Banding patterns determined by PFGE of 16 isolates of *K. pneumoniae* were divergent except for the isolate number 3 and 5, which were clonally indistinguishable

Compared to the PCR method, which served as a reference, both the capsular swelling and strip tests yielded consistent results on identification of serotypes K1 and K2. Results on serotypes K1 and K2 from different methods are presented in Fig. 1a–c. Among the 16 *K. pneumoniae* isolates from liver abscesses, 50.0% (8/16) turned out to have serotype K1, 18.8% (3/16) serotype K2, and 31.3% (5/16) non-K1/K2 serotype. The five *K. pneumoniae* non-K1/K2 serotypes were K55, K7, K53, K14, and K57 as confirmed by the capsular swelling test. In eight patients with K1 *K. pneumoniae* liver abscesses, two patients with complications of distant metastatic endophthalmitis according to clinical findings (posterior or anterior ocular inflammation via funduscopic examination) and radiological studies. Sixteen strains from patients with *K. pneumoniae* liver abscesses were analyzed via pulse-field gel electrophoresis (PFGE). Most strains had distinct PFGE patterns except for the isolate number 3 and 5, which were clonally indistinguishable (Fig. 2).

### ICS analysis of positive flagged blood culture broth samples and pure bacterial culture

A total of 112 blood culture broth samples with positive signals for microorganisms and subsequent pure bacterial colony growth on solid culture media were included in ICS efficacy testing. Among the 108 *K. pneumoniae* samples, testing of blood culture broth samples with ICS revealed that 14 belonged to serotype K1 and 16 belonged to serotype K2. Testing for a single colony with ICS produced consistent results for most samples. Different results between tests of blood culture broth samples and tests of a single colony were obtained, indicating that two non-K1/K2 results from blood broth samples were identified as the K2 serotype in the pure single colony. Subsequent PCR typing of the two isolates with discrepant results revealed that all of them were of the K5 serotype. For the four non-*K. pneumoniae* samples from blood culture broth samples and pure bacterial culture (*Staphylococcus capitis, S. aureus, Proteus mirabilis, and Pseudomonas aeruginosa*), negative results were produced by the ICS kit (Table 2).

**Table 2:** ICS test results on 108 *K. pneumoniae* isolates from blood culture bottles and pure colonies

Results of strip	Blood culture broth			Single colony		
	K1(+)	K2(+)	Negative	K1(+)	K2(+)	Negative
Case Number	14	16^a^	78^a^	14	18	76

*ICS* immunochromatographic strip

^a^Two non-K1/K2 isolates from blood culture broth samples were identified as the K2 serotype in subsequent single colony broth samples and further serotyping by PCR confirmed serotype K5

## Discussion

Capsular polysaccharide has been shown to be an important virulent factor of *K. pneumoniae* infection pathogenicity [8]. Among the 79 recognized capsular serotypes, K1 and K2 have been reported to have a significant association with increased virulence and septicemia-related infection rates, as well as liver abscesses associated with distant metastasis, including endophthalmitis [5]. In a recent study out of a French intensive care unit, hypervirulent *K. pneumoniae* infections were found to result mostly from community-acquired infections and were predominately caused by *K. pneumoniae* serotypes K1 and K2. Although mortality rates between hypervirulent and nonhypervirulent *K. pneumoniae* strains were not different, these community-acquired hypervirulent *K. pneumoniae* strains yielded higher rates of multiorgan failure [9]. In addition, antiphagocytosis activity and serum resistance to bactericidal activity for *K. pneumoniae* with capsular serotype K1 and K2 antigens have been reported regarding clinical *K. pneumoniae* infections [4].

In vitro and in vivo studies have revealed that serotypes K1 and K2 cause an increase in disease lethality [10]. According to an epidemiological study on *K. pneumoniae*-related liver abscesses in endemic regions, most cases are monomicrobial infections [11–13]. Some studies have shown that the strains from community-acquired liver abscesses are highly susceptible to cefazolin and other extended-spectrum antibiotics, including cephalosporins [3, 14]. The susceptibility of *K. pneumoniae* in liver abscesses has remained unchanged even in recurrent *K. pneumoniae* liver abscesses [15, 16]. Because broad-spectrum antimicrobials combined with earlier abscess drainage for patients was recommended [15], rapid detection of the infection could help to confirm the appropriate regimen for these patients [1], resulting in a reduction in medical costs. This way, shorter hospital stay in addition to lower antibiotic consumption could be achieved. In addition, early K1 and K2 detection will alert physicians to possible distant metastatic infectious complications such as endophthalmitis. In our study, there were no deaths after the rapid identification of K1 and K2 capsular serotypes, with the administration of adequate antibiotics. Two patients with distant endophthalmitis-associated metastases with serotype K1 identified by ICSs had a good prognosis due to quick management with systemic antibiotics and local treatment.

Apart from direct pus analysis after drainage, ICS application to a positive blood culture medium yielded matching results, when compared to pure bacterial cultures. In clinical microbiology laboratories, blood culture shows positive signals for microorganisms, and the process usually takes approximately one more day for subculture formation and identification. Using the ICSs, it is possible to rapidly identify *K. pneumoniae* serotypes K1 and K2 in samples with a high probability of *K. pneumoniae* bacteremia in an endemic area, such as in liver abscess cases in Southeast Asia [17, 18]. The ICSs may be valuable for selecting the antimicrobial therapy in spite of broad antimicrobial coverage for bacteremia cases. Nonetheless, in view of the various pathogens that may be cultured in blood culture systems, the drawback of the ICS application to positive blood culture samples is a high negative rate, because ICS cannot detect all *K. pneumoniae* bacteremia strains, only serotypes K1 and K2. This approach should be more helpful if a new ICS kit which includes detection of additional specific *K. pneumoniae* strains could be developed. Although our results revealed a high concordance rate for testing of positive blood culture samples and isolates, there was a discrepant result. We suspect that the discrepancy resulted from the nutritious blood culture broth which contains many substances which might interfere with the antigen–antibody reaction.

Among all the various serotypes of *K. pneumoniae* used to assess the specificity of the ICS kit in previous studies, there were no false positive or false negative results produced by the ICS kit, and these data were comparable to PCR and serum agglutination results [7]. In the present study, the color reactions for pus in both the control and the test lines were seen within 3 to 4 min for both serotypes K1 and K2. Positive color reactions for the control line and negative reactions for the test line in our test were obtained for *K. pneumoniae* serotypes other than K1 and K2, which were later demonstrated to be serotypes K7, K14, K53, K55, and K57. PFGE findings revealed that most strains tested in the current study were unrelated sporadic isolates. After the comparison of the ICSs to other conventional test methods—PCR and serum agglutination—the results were consistent. This suggested that ICSs have high specificity, equal to that of other methods on different clinical *K. pneumoniae* strains..

Traditional serotyping methods include serum agglutination, countercurrent immunoelectrophoresis, and PCR, which have been the standard methods for confirming *K. pneumoniae* capsular serotypes [2, 4, 5, 7, 19, 20]. Nevertheless, these methods require specific materials such as larger doses of anticapsular serum, primers, chemical mixes, and gel preparations in addition to well-qualified technicians and equipment. This situation can cause delays with the results, not to mention the complicated procedure. Although PCR is a more sensitive test with a very low detection limit, it took 4 h. Unlike conventional detection methods, the rapid ICS method does not require skilled technicians or special equipment. In fact, it is extremely easy to use because it requires only simple visual analysis. Moreover, ICS specificity is comparable to that of other methods. The advantage of the ICS method described here is that ICSs do not require significant preparation before testing the pus directly after ultrasonography-guided or computed tomography (CT)-guided aspiration.

## Conclusions

In summary, this study revealed that ICSs are highly specific and sensitive for the direct detection of *K. pneumoniae* serotypes K1 and K2 in pus and samples of positive blood culture broth in clinical settings, as compared with serum agglutination with countercurrent immunoelectrophoresis and PCR from a culture colony. ICSs can serve as a rapid tool for the identification of hyper-virulent *K. pneumoniae* strains that can cause a liver abscess.

## Methods

### Patients

The study was conducted at the Tri-Service General Hospital, a teaching hospital with 1800 beds in northern Taiwan and at Kaohsiung Medical University Hospital with 1600 beds in southern Taiwan from July 2017 to November 2017. Patients with a pyogenic liver abscess as confirmed by abdominal sonography or abdominal CT scans who underwent an ultrasonography- or CT-guided aspiration of an abscess, were enrolled. Clinical data were retrospectively reviewed.

### Collection of *K.* *pneumoniae* with different serotypes and sites of isolation

During the study period, *K. pneumoniae* strains from liver abscesses were collected for capsular serotyping by the capsular swelling test, PCR, and ICSs. We also collected both blood broth samples from positive blood culture bottles and the subsequent pure bacterial colony grown on solid media for capsular serotyping with ICSs. Bacterial identification was performed by matrix-assisted laser desorption/ionization time-of-flight mass spectrometry (bioMérieux, France).

### The traditional capsular swelling test for capsular serotypes

*Klebsiella pneumoniae* strains isolated from pyogenic liver abscesses were subjected to the traditional capsular serotype test. Serotyping was conducted by the capsular swelling technique after inoculation of agar and incubation at 37 °C [2, 4]. Control *K.* *pneumoniae* serotypes, including ATCC4208 (K1), were acquired from the American Type Culture Collection (ATCC, Rockville, MD).

### PCR for capsular serotyping

*Klebsiella pneumoniae* isolates from liver abscesses were genotyped for serotypes K1 and K2 by PCR as previously described and were served a reference to compare with the capsular swelling test and ICS results [5, 19, 20]. PCR was carried out to determine the presence of the specific genes for serotypes K1 and K2. The primers specific for K1 consisted of the oligos 5′-GGTGCTCTTTACATCATTGC-3′ and 5′-GCAATGGCCATTTGCGTTAG-3′. K2 primers were 5′-GACCCGATATTCATACTTGACAGAG-3′ and 5′-CCTGAAGTAAAATCGTAAATAGATGGC-3′. Thermocycler conditions were: 1 cycle of denaturation at 94 °C for 1 min, followed by 30 cycles of 94 °C for 30 s, annealing at 58 °C for 45 s and elongation 72 °C for 1 min 30 s and a final extension at 72 °C for 10 min. The amplification was visualised by electrophoresis using 1.5% agarose gel followed by staining in ethidium-bromide.

### ICS analysis of the capsular serotype

**Fig. 3:**
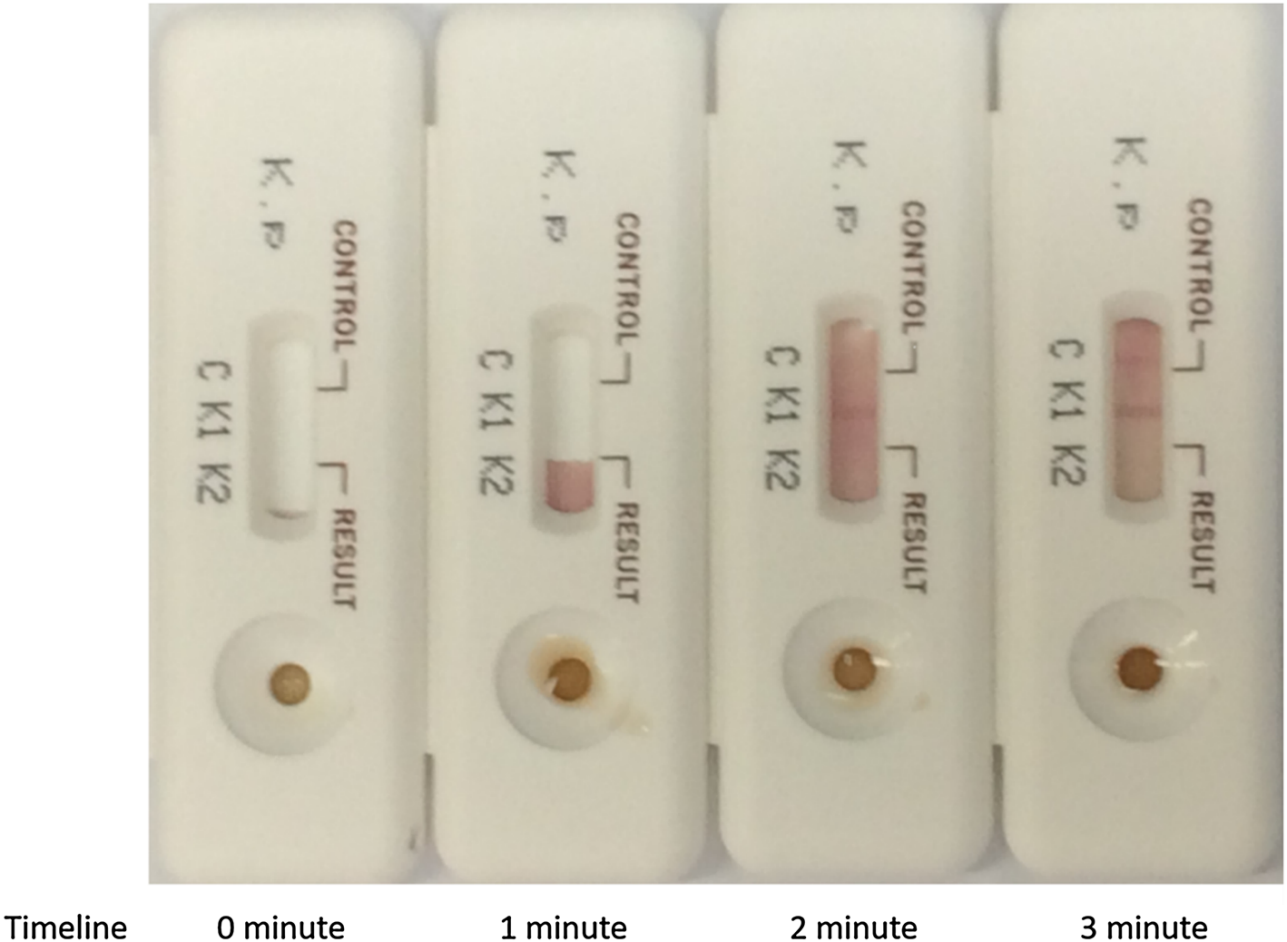
Identification process for serotype K1 *K. pneumoniae* using ICSs. After the sample was added to the sample loading zone, the red line of serotype K1 (K1) is shown at the second minute. The line of serotype K2 (K2) is not visible in the second minute, and the control line (C) developed in the third minute. Serotype K1 was identified within 3 min. Timeline is indicated at the bottom

The ICSs were prepared by KeMyth Biotech (Taipei, Taiwan) which had been described before [7]. According to previous study, the ICSs could quickly identify serotypes K1 and K2 *K. pneumoniae* isolates from single bacterial colony within 5 min. No false-positive or false-negative results were ever seen when testing various serotypes of *K. pneumoniae* along with other bacterial species. In addition, the ICSs can be stored at room temperature for at least 6 months without losing their sensitivity or specificity [7]. In this study, directly aspirated pus from patients was obtained after an ultrasonography- or CT-guided aspiration procedure. The pus was added into the sample-loading well of the test cassette. If the sample was too mucoid, sterile injection water was mixed with the pus in a 4:1 ratio. Results were read a few minutes later, and this period was chosen as the cut-off point to determine the results. A result obtained after 5 min was considered invalid. In Fig. 3, we demonstrate the procedure and timing for testing by means of ICSs when detecting *K. pneumoniae* serotype K1.

### PFGE

PFGE typing was performed for 16 *K. pneumoniae* isolates from pus samples. Total DNA was prepared and digested with XbaI (New England Biolabs Beverly, MA, USA) according to the manufacturer. The restriction fragments were loaded into a 1% agarose gel (Bio-Rad, Hercules, CA, USA) in the running buffer, 0.5 × TBE buffer (45 mM Tris, 45 mM boric acid, and 1.0 mM EDTA [pH 8.0]) with a CHEF Mapper apparatus (Bio-Rad Laboratories, Richmond, CA, USA). The electrophoresis conditions were at 200 V for 24 h, with pulse times of 2–40 s. Gels were stained with ethidium bromide and photographed under UV light.

### Capsular serotype detection by ICSs in positive blood culture broth samples and pure bacterial culture

A total of 108 positive flagged blood culture broth samples identified as *K. pneumoniae* via matrix-assisted laser desorption/ionization time-of-flight mass spectrometry and a subsequent pure bacterial colony grown on solid culture media were tested with ICSs for comparison. Besides, another 4 positive flagged blood culture samples caused by bacteria other than *K. pneumoniae* (2 Gram-positive and 2 Gram-negative microorganisms) were randomly chosen and tested with ICS also. From each blood culture bottle, 120 µl of broth was loaded onto the ICS cassette, and the result was read after 3 min. For pure bacterial culture testing, a single colony of the grown isolate from the blood culture was mixed with 500 µl of sterile saline; 120 µl of the suspension was loaded onto the ICS cassette, and the results were read after 3 min. When inconsistent results were obtained in the comparison of blood culture broth samples with pure bacteria samples by means of ICSs, the wzi gene sequencing method for serotype confirmation was employed [21].

## Abbreviations

ICSs: immunochromatographic strips
PCR: polymerase chain reaction
PFGE: pulse-field gel electrophoresis
CT: computed tomography
